# Fear of COVID-19 and workplace phobia among Pakistani doctors: A survey study

**DOI:** 10.1186/s12889-021-10873-y

**Published:** 2021-04-30

**Authors:** Sadia Malik, Irfan Ullah, Muhammad Irfan, Daniel Kwasi Ahorsu, Chung-Ying Lin, Amir H. Pakpour, Mark D. Griffiths, Ibad Ur Rehman, Rafia Minhas

**Affiliations:** 1 Department of Psychology, University of Sargodha, Sargodha, Pakistan; 2 Kabir Medical College, Gandhara University, Peshawar, 25000 Pakistan; 3 Internal Medicine, Hayatabad Medical Complex, Peshawar, Pakistan; 4 Department of Rehabilitation Sciences, Faculty of Health & Social Sciences, The Hong Kong Polytechnic University, Hung Hom, RM QT 512 Hong Kong China; 5 Institute of Allied Health Sciences, College of Medicine, National Cheng Kung University, Tainan, Taiwan; 6 Social Determinants of Health Research Center, Research Institute for Prevention of Non-Communicable Diseases, Qazvin University of Medical Sciences, ShahidBahounar BLV, Qazvin, 3419759811 Iran; 7 Department of Nursing, School of Health and Welfare, Jönköping University, Jönköping, Sweden; 8 International Gaming Research Unit, Psychology Department, Nottingham Trent University, 50 Shakespeare Street, Nottingham, NG1 4FQ UK; 9 Shifa College of Medicine, Shifa Tameer-e-Millat University, Islamabad, Pakistan; 10 Avicenna Medical and Dental College, Lahore, Pakistan

**Keywords:** Fear of COVID-19, Workplace phobia, Doctors, Workplace panic anxiety, Avoidance behavior

## Abstract

**Background:**

The novel coronavirus disease-2019 (COVID-19) has seriously affected the lives of millions of people across the world. It has also heavily burdened healthcare professionals and the virus poses serious risks for their personal and professional lives. Therefore, the present study examined the associations between fear of COVID-19 and workplace phobia among doctors in Pakistan during the COVID-19 pandemic.

**Methods:**

An online survey was conducted among 421 doctors in Pakistan between April 10 and May 25, 2020. The Workplace Phobia Scale (WPS) and the Fear of COVID-19 Scale (FCV-19S) were the main psychometric instruments used in this study.

**Results:**

There was a significant positive relationship between fear of COVID-19 and workplace panic anxiety and workplace avoidance behavior. Significantly higher fear of COVID-19 was found among (i) females compared to males, (ii) doctors with 5 years or less of work experience compared to those with more than 5 years, and (iii) postgraduate trainees compared with other ranks. Two groups (doctors who were above 30 years old and postgraduate trainees) were found to have higher levels of workplace phobia compared to their counterparts. Doctors with severe levels of fear of COVID-19 had significantly higher levels of workplace panic anxiety and workplace avoidance behavior.

**Conclusions:**

Fear of COVID-19 was significantly associated with workplace phobia which may negatively affect doctors’ performance. Therefore, important steps are needed to protect doctors’ health by providing sufficient resources to allay their fears and anxieties which consequently help them in carrying out their frontline duties in response to the COVID-19 pandemic.

## Background

The world is facing a unique challenge due to the emergence of the novel coronavirus disease-2019 (COVID-19) which has spread to a majority of countries worldwide after the initial outbreak in Wuhan, China [1]. Initially, the response to the emergence of the virus was relatively slow but after World Health Organization (WHO) declared COVID-19 as a pandemic, most countries implemented specific policies such as home-confinement, physical distancing, wearing of masks, frequent washing of hands, and in extreme cases, nationwide lockdowns for preventing, treating, and containing the spread of COVID-19 [2–4]. The disease has been associated with increased anxiety and fear among individuals worldwide [5–7]. These responses are natural considering the dangerous and unpredictable nature of the virus [5–7].

Individuals infected with SARS-COV-2 show symptoms of fever, fatigue, dry cough, myalgia, and dyspnea within first 2 weeks of contact [8]. By March 1, 2020, the mortality rate of COVID-19 had reached 3.6% in China and 1.5% in other countries [9]. According to a previous study, among communicable diseases, lower respiratory tract infections have the highest mortality globally [10]. The morbidity and mortality associated with COVID-19 during the pandemic have raised many problems in various sectors. More specifically, it has been claimed that mental health issues faced by medical staff are the least recognized and under-addressed [11]. However, healthcare professionals’ mental health needs should be recognized because they are not exempted from experiencing them. According to a study conducted in Pakistan among trainee doctors during the COVID-19 pandemic, the prevalence of depressive symptoms, generalized anxiety disorder, and acute stress disorder were 26.4, 22.6, and 4.4%, respectively [12]. Another study among doctors during the pandemic from Lahore (Pakistan) reported severe anxiety (7.2%) and depression (1%) among a minority of those surveyed [13]. These Pakistani studies are important to because they are in the same country setting as the present study. Therefore, findings may help with understanding the mental health challenges among Pakistani doctors.

Previous studies on severe acute respiratory syndrome (SARS) which has similarities to that of COVID-19 suggested that healthcare workers (HCWs) and especially doctors were specifically more prone to developing anxiety, depression, and stress [14]. This may be possible based on the effect of either contracting the virus or the work demands. This is because they are at a greater risk of contracting the disease due to their direct involvement in screening and treating SARS. Therefore, if an HCW becomes infected with SARS-COV-2, the sudden reversal of role from HCW to a patient adds to the levels of fear, frustration, stigma, and adjustment issues [15]. On the other hand, the long duty shifts, increased work hours, lack of personal protective equipment (PPE), and lack of social support due to COVID-19 may affect their mental health [16, 17]. As aforementioned, the lack of appropriate PPE, heavy workloads [18], hostile environment and confrontation [19] may severely affect frontline workers and lead to workplace phobia [20]. This may explain why a worker might find it difficult to function effectively in the workplace and use avoidance behaviors which in turn may lead to high levels of anxiety [21].

Workplace phobia has been a common problem in primary healthcare [22] but with the current COVID-19 pandemic and frontline workers being directly affected, workplace-related anxiety might be even more prevalent [15]. However, the association between COVID-19 and workplace phobia has been understudied and needs further research since it is highly applicable in the current pandemic [23].

The dangerous and unpredictable nature of COVID-19 has instilled fears among doctors worldwide and especially among Pakistani doctors [19, 24]. As of April 25, 2021, there had been over 795,000 confirmed COVID-19 cases and over 17,000 deaths in Pakistan [25]. A previous study has shown that an infectious disease such as COVID-19 with its high transmission rate and relatively high mortality rate can lead to psychosocial problems including stigmatization and bias on some level [26]. Furthermore, doctors were also afraid of transmitting the virus to their families as well as getting infected themselves. With no proven cure when the data were collected, insufficient PPE, a lack of a nurturing and accommodating environment, and a lack of support from concerned health authorities, doctors may develop anxieties, fear and/or aversion towards their duties [1, 18, 19, 24]. If the level of fear of COVID-19 were to exceed a specific limit, it might even affect doctor’s rationality and decision-making ability and detrimentally impact their work [27]. Therefore, there is the need for further research to appropriately address the expectation and fears of doctors in these unprecedented times.

The maintenance of healthy psychological wellbeing among doctors is important to consider because they are at the heart of the healthcare system and there is a current lack of knowledge regarding how fear debilitates their mental health capacity. Based on the aforementioned background literature, the present study examined the associations between fear of COVID-19 and workplace phobia among doctors in Pakistan during the COVID-19 pandemic. More specifically, this study examined the (i) relationship between fear of COVID-19 and workplace panic anxiety and workplace avoidance behavior, (ii) between-group differences on fear of COVID-19 and workplace phobia, and (iii) differences between the different severity levels of fear of COVID-19 among doctors on their workplace phobia (i.e., workplace panic anxiety and workplace avoidance behavior). Through these results, we hope to shed light on Pakistani doctors’ mental health during COVID-19, so that mental health professionals can further assist their needs.

## Methods

### Participants and design

This cross-sectional study was conducted among doctors (*N* = 421) dealing with COVID-19 patients in various Pakistani hospitals from April 10 to May 25, 2020. Ethical approval was obtained from the Institutional Review Board (IRB) at the University of Sargodha (Pakistan). A convenience sampling method was used to recruit participants who were available and willing to participate in the study by completing an online survey. To avoid sampling errors, we took different steps. We requested access to doctors working in COVID-19 wards and isolation centers from the hospital administration. The survey link was shared among the doctors dealing with COVID-19 patients through WhatsApp and Facebook groups of Pakistani doctors either directly or indirectly through hospital administrators. Also, the selection criteria (e.g., doctors treating COVID-19 patients) were made very clear to the doctors as well as including salient information (i.e., demographic details, city, COVID-19 ward or/and isolation center) in the survey about their work. Informed consent was obtained from all participants and they were assured that the information they provide would be anonymous and confidential, and only used for research purposes. All demographic information about the participants can be found in Table 1.

**Table 1:** Demographic characteristics of the participants (*N* = 421)

	Number of participants	Percentage (%)
*Gender*		
Male	285	67.7
Female	136	32.3
*Age*		
≤ 30 years	271	64.4
> 30 years	150	35.6
*Marital status*		
Single/divorced	237	56.3
Married	184	43.7
*Sector*		
Government	365	86.7
Private	56	13.3
*Rank*		
Consultant	60	14.3
House officers	51	12.1
Medical officers	125	29.7
Postgraduate trainees	185	43.9
*Department*		
Medicine	135	32.1
Surgery	50	11.9
Gynecology	44	10.5
Pediatrics	36	8.6
Emergency	21	5.0
ENT	20	4.8
Ophthalmology	15	3.6
Anesthesia	6	1.4
Cardiology	9	2.1
ICU	9	2.1
Gastroenterology	7	1.7
Dermatology	7	1.7
Dentistry	6	1.4
Radiology	6	1.4
Nephrology	6	1.4
Pulmonology	6	1.4
Other	38	9.0
*Experience*		
≤ 5 years	356	84.6
> 5 years	65	15.4

*ENT* Ear, Nose, Throat department; *ICU* Intensive Care Unit

### Measures

The survey comprised three sections which are the (i) socio-demographic characteristics, (ii) workplace phobia, and (iii) fear of COVID-19. To assess workplace panic anxiety and avoidance behavior, the Workplace Phobia Scale was used. To assess fear of COVID-19, the Fear of COVID-19 Scale (FCV-19S) was used. English versions of the scales were used as it is the language used for teaching medical professionals and also for writing clinical notes in Pakistani hospitals. Also, the reliability of the scales as used in the English language were tested and all the scales showed acceptable psychometric properties. Details about the scales and their psychometric properties are provided below.

#### Workplace phobia scale (WPS)

The WPS is a 13-item self-report scale that assesses workplace panic anxiety and avoidance behaviors [28, 29]. The workplace panic anxiety subscale assesses physical panic-like reactions when individuals think about the workplace while workplace avoidance behavior assesses avoidance behavior that individuals show towards the workplace. The items (e.g., “*When thinking about my workplace, everything in my body is tense”*) are rated on a five-point scale from 0 (*absolutely disagree*) to 4 (*absolutely agree*) with total scores ranging from 0 to 52. Higher scores indicate higher levels of workplace phobia. The reliability of the scale in the present study was excellent (Cronbach’s alpha = .938).

#### Fear of COVID-19 scale (FCV-19S)

The FCV-19S is a 7-item uni-dimensional self-report scale that assesses the fear of COVID-19 [27]. The items (e.g., “*I am afraid of losing my life because of coronavirus-19”*) are rated on a five-point scale from 1 (*strongly disagree*) to 5 (*strongly agree*) with total scores ranging from 7 to 35. The higher the score, the higher the level of fear of COVID-19. The FCV-19S was classified into mild (score ≤ 14), moderate (score 14 to 28) and severe (score ≥ 28) level based on a mean-standard deviation formula similar to a previous study [30]. The scale has been validated among Pakistanis [31]. The reliability of the scale in the present study was excellent (Cronbach’s alpha = .908).

### Data analysis

SPSS software, version 21, was used for analyzing the data. Means and percentages (where appropriate) for each variable were calculated. Categorical variables were analyzed using chi-square tests or Fisher’s exact test. Continuous variables were analyzed using independent *t*-tests, a one-way analysis of variance (ANOVA) for between-group differences, and Pearson *r* for the relationship between the variables. A multivariate regression analysis was then performed to identify factors associated with fear of COVID-19 and workplace phobia after the assumptions were met. The basic assumptions of the regression analysis were confirmed before conducting the analysis. To guarantee the normality of data, skewness and kurtosis were computed. The value of tolerance and variance inflation factor showed that multicollinearity was not a serious problem. After the confirmations, the main analyses were carried out. Differences between groups were considered to be significant when the *p-*value was < .05.

## Results

A total of 421 doctors (285 males, 67.7%; 136 females, 32.3%) with a mean age of 30.81 (*SD* = 6.22) years participated in the study. Over half the participants were single (56.3%). Most worked in the public sector (86.7%), while the remainder worked in the private sector (13.3%). The sample comprised 14.3% consultants, 12% house officers, 29.7% medical officers, and 43.9% postgraduate trainees. Most participants (84.6%) had five or more years of experience in their field (Table 1).

The results of bivariate correlations in Table 2 showed that there was a significant positive correlation between fear of COVID-19 and workplace phobia in general (*r* = .72, *p* < .01) and between the subscales; (ii) fear of COVID-19 and workplace phobic panic anxiety (*r* = .74, *p* < .01), and (iii) fear of COVID-19 and workplace avoidant behavior (*r* = .64, *p* < .01).

**Table 2:** Relationship between fear of COVID-19, anxiety and work phobic anxiety

		1	2	3	4
1	Fear of COVID-19	–	.718**	.736**	.638**
2	Work phobic anxiety		–	.974**	.955**
3	Work phobic panic anxiety			–	.865^**^
4	Work phobic avoidance anxiety				–

**p < .01

Table 3, a regression analysis, shows that fear of COVID-19 predicted workplace phobic anxiety among doctors. Specifically, fear of COVID-19 significantly predicted workplace panic anxiety (standardized coefficient [*β*] = .617, *p* < .001) and workplace avoidance behavior (*β* = .546, *p* < .001). The value of ∆R^2^ of .380 and .296 indicated that among doctors, fear predicted 38% variance in workplace panic anxiety and 29.6% in workplace avoidance behavior.

**Table 3:** Predicting role of fear of COVID-19 on workplace phobia among doctors

	Workplace panic anxiety			Workplace avoidance behavior		
Variable	∆R^2^	β	F	∆R^2^	β	F
Fear of COVID-19	.380	.617***	257.94***	.296	.546***	177.56***

***p < .001

As shown in Table 4, female doctors (*M* = 22.18, *SD* = 6.9) had significantly higher fear of COVID-19 scores (*t*(419) = − 2.531, *p* = .012) compared to male doctors (*M* = 20.32, *SD* = 7.05). Furthermore, the results showed that postgraduate trainee doctors (*M* = 22.75, *SD* = 6.66; *M* = 23.15, *SD* = 13.20 for fear of COVID-19 and workplace phobia respectively) had significantly higher fear of COVID-19 (*F*(3, 417) = 8.285, *p* < .001) and workplace phobia (*F*(3, 417) = 4.574, *p* = .004) as compared to house officers (*M* = 20.02, *SD* = 6.72; *M* = 20.06, *SD* = 11.94), medical officers (*M* = 18.96, *SD* = 6.93; *M* = 17.66, *SD* = 12.94), and consultants (*M* = 20.15, *SD* = 6.76; *M* = 20.72, *SD* = 12.47). Finally, the results demonstrated that doctors with less than 5 years’ experience (*M* = 21.29, *SD* = 7.00) had significantly higher fear of COVID-19 (*t*(419) *=* 2.481, *p* = 013) compared to those who had more than 5 years’ experience (*M* = 18.94, *SD* = 7.09) in their field. Experience as a trainee did not count as *experience*. Therefore, trainees were deemed as having no years of experience in the present study.

**Table 4:** Between-group comparison on fear of COVID-19 and workplace phobia

	Fear of COVID-19		Work phobia	
	*M (SD)*	*p*-value	*M (SD)*	*p*-value
*Gender*				
Male	20.32 (7.05)	.012*	20.27 (12.72)	.230
Female	22.18 (6.95)		21.90 (13.66)	
*Age*				
≤ 30 years	20.70 (7.11)	.385	19.71 (13.01)	.034*
> 30 years	21.33 (6.99)		22.61 (12.94)	
*Marital status*				
Single/divorced	21.18 (7.25)	.42	20.66 (13.41)	.78
Married	20.62 (6.86)		21.01 (12.65)	
*Occupational sector*				
Government	20.91(6.99)	.899	20.80 (12.97)	.985
Private	21.04 (7.57)		20.77 (13.60)	
*Rank*				
Consultant	20.15 (6.76)	.000***	20.72 (12.47)	.004**
House officers	20.02 (6.72)		20.06 (11.94)	
Medical officers	18.96 (6.93)		17.66 (12.94)	
Postgraduate trainees	22.75 (6.66)		23.15 (13.20)	
*Work experience*				
≤ 5 years	21.29 (7.00)	.013*	20.96 (12.96)	.557
> 5 years	18.94 (7.09)		19.91 (13.54)	

*p < .05; **p < .01; ***p < .001. *M* and *SD* represent *Mean* and *Standard Deviation* respectively

Table 5 shows the differences between the different severity levels of fear of COVID-19 among doctors in relation to their workplace phobia, and more specifically workplace panic anxiety and workplace avoidance behavior. Findings indicated that doctors with a severe level of fear of COVID-19 (*M* = 20.42, *SD* = 4.88; *M* = 14.12, *SD* = 4.55) showed significantly more workplace panic anxiety (*F*(2, 418) = 130.12, *p* < .001) and avoidance behavior (*F*(2, 418) = 92.42, *p* < .001) compared to those having mild (*M* = 4.77, *SD* = 4.86; *M* = 3.67, *SD* = 3.97) and moderate (*M* = 11.77, *SD* = 6.59; *M* = 7.75, *SD* = 5.15) levels of fear of COVID-19.

**Table 5:** Severity levels of fear of COVID-19 among doctors on their workplace phobia

Category	***N***	***M***	***SD***	***F***	***p***	LL	UL	Post Hoc
*Workplace panic anxiety*								
Mild fear^1^	67	4.77	4.86	130.124	< .001	3.59	5.96	3 > 2 > 1
Moderate fear^2^	285	11.77	6.59			10.98	12.56	
Severe fear^3^	85	20.42	4.88			19.37	21.48	
*Workplace avoidance behavior*								
Mild fear^1^	67	3.67	3.97	92.423	< .001	2.71	4.65	3 > 2 > 1
Moderate fear^2^	285	7.75	5.15			7.14	8.37	
Severe fear^3^	85	14.12	4.55			13.13	15.09	

*N* Number of participants, *M* Mean, *SD* Standard Deviation, LL Lower Limit, UL Upper Limit

## Discussion

The present study examined the fear of COVID-19 and workplace phobia among Pakistani doctors. More specifically, it examined the (i) relationship between fear of COVID-19 and workplace panic anxiety and workplace avoidance behavior, (ii) between-group differences on fear of COVID-19 and workplace phobia, and (iii) differences between the different severity levels of fear of COVID-19 among doctors on their workplace phobia (i.e., workplace panic anxiety and workplace avoidance behavior). The findings showed that fear of COVID-19 was significantly associated with workplace panic anxiety and avoidance behavior. That is, there was a significant positive relationship between fear of COVID-19 and workplace phobia as well as its subscales (workplace panic anxiety and workplace avoidance behavior). All the relationships had a large effect [32, 33]. This indicates that the higher the doctor’s fear of COVID-19 then the higher their workplace phobia (in general) and specifically workplace panic anxiety and workplace avoidance behavior may also be. If these persist without efficient coping or supportive system or environment, may eventually affect their performance at work. Recent studies have shown that fear of COVID-19 has impacted negatively on individuals’ occupational lives [34–36]. During the COVID-19 pandemic, the mental health of the professional healthcare workforce has noticeably been affected.

Furthermore, the present study found that fear of COVID-19 was a significant predictor of workplace panic anxiety and workplace avoidance behavior among doctors. Fear of COVID-19 accounted for 38% variance in workplace panic anxiety and 29.6% in workplace avoidance behavior among doctors. This indicates that the fear of COVID-19 has the potential of negatively affecting doctors work by specifically triggering phobic reactions (i.e., panic anxiety or avoidance behavior). Therefore, it is important that sufficient resources and enabling working conditions are provided for the doctors to allay their fears of COVID-19 infection. Previous studies have shown that fear of infection has a negative psychological effect on health professionals who take care of patients during epidemics which supports the present study’s findings among doctors [5, 11–13].

It was further found that females, postgraduate trainees, and those with five or less years of working experience had significantly higher levels of fear of COVID-19 compared to their comparative counterparts. This indicates that females, postgraduate trainees, and those with five or less years of working experience, compared to their comparative counterparts, may need extra resources or support to allay their fear of COVID-19. A possible explanation for this result may be that trainees are new to the job, so they have not had as much exposure to highly infectious diseases and the pandemic may be more distressing for them hence, the higher levels of fear of COVID-19 than their seniors. This also applied to doctors with 5 years or less working experience. These findings are consistent with those of Temsah et al. [37] who reported that doctors were so overwhelmed with the fear of COVID-19 that 15% of doctors considered rescheduling or changing their duty so that they could avoid COVID-19 patients. Similarly, Zhang et al. [38] reported that during the COVID-19 pandemic, female medical health workers experienced greater fear, anxiety, depression, and obsessive-compulsive symptoms than male doctors. Also, two groups (doctors who were more than 30 years old and postgraduate trainees) had significantly higher levels of workplace phobia compared to their comparative counterparts suggesting that they may have panic anxiety or avoid work compared to their respective counterparts. Postgraduate trainees may need special attention in the form of mentorship and counseling or psychotherapy to deal with their challenges because they had higher levels of fear of COVID-19 and then workplace phobia [39, 40]. Possible reasons for doctors older than 30 years may be that they worry about COVID-19 infection transmission from the workplace to their families or job security after COVID-19 infection [36, 41]. Nonetheless, hospital administration or authorities must urgently refer such individuals to mental health officers for further help.

In addition, it was found that there were differences between the different severity levels of fear of COVID-19 on workplace panic anxiety and avoidance behavior. More specifically, doctors with a severe level of fear of COVID-19 showed significantly more workplace panic anxiety and avoidance behavior compared to those having mild and moderate levels of fear of COVID-19. This supports the earlier assertion that higher levels of fear of COVID-19 are associated with workplace panic anxiety and avoidance behavior and by extension, other mental health challenges. The findings of the present study are consistent with previous studies in Japan and Singapore also reported a high level of fear and anxiety among HCWs during the SARS-CoV outbreaks [42, 43] and may also underlie the reasons for the higher mental health challenges among Pakistani doctors [12, 13].

The present study has some specific limitations. First, the convenience sampling method was used to recruit the doctors and so may not represent the total Pakistani doctor population. Future studies should carry out such a survey on a nationally representative sample to confirm the findings of this study. Second, the present study used a cross-sectional design which by nature can only show the association between variables. Therefore, longitudinal studies are needed to determine causality between the study’s variables. Therefore, researchers should interpret the study’s findings with caution. Third, the sample was not equally distributed with respect to gender which may have affected the findings. Fourth, this study merged single and divorced categories but there may be differences in these two groups. Therefore, future studies may want to separate these relationship categories so as to examine their unique differences. Finally, all the data were self-report and are therefore subject to established methodological biases.

Healthcare workers can experience a serious risk of COVID-19 exposure and consequently experience different detrimental psychological conditions, lack of sleep, low job satisfaction, fear, and work-related anxiety [36, 44]. The present study demonstrated significant associations between fear of COVID-19 and workplace phobia (i.e., workplace panics anxiety and workplace avoidance behavior). Therefore, it is important to provide doctors with adequate personal protective equipment (PPE) and a proper work-rotation schedule. The findings of the present study have significant clinical and public health implications. First, as the samples were doctors, the findings could be helpful to clinicians, organizational psychologists, and health administrators in understanding how work-related pressures can negatively influence the mental health of the HCW. The findings could also help in the design of psychological interventions to deal with such unprecedented pressures and its related psychological outcomes. As doctors and other healthcare workers play an important and frontline role in the war against COVID-19, it is necessary to ensure their good mental health by encouraging them to express their thoughts and by providing psychological help and talk therapies. The findings of this study may also help understand the needs of doctors in relation to proper training and guidance regarding Standard operating procedure (SOPs), dealing with patients, and preventive measures to deal with COVID-19. It is recommended that adequate PPE and training should be provided to the doctors in dealing with COVID-19 patients. Clear instructions regarding diagnosis and treatment protocol will help relieve work pressure and increase occupational satisfaction because it will help doctors in Pakistan to understand the severity of the problem and how to cope up with it.

## Conclusion

The present study examined the associations between fear of COVID-19 and workplace phobia among doctors in Pakistan during the COVID-19 pandemic. It was found that there were significant positive associations between fear of COVID-19 and workplace phobia (i.e., workplace panic anxiety and workplace avoidance behavior). Also, females, postgraduate trainees, and those with five or less years of working experience had significantly higher levels of fear of COVID-19 compared to their counterparts. Furthermore, two groups (doctors who were more than 30 years old and postgraduate trainees) had significantly higher levels of workplace phobia compared to their counterparts. Additionally, doctors with severe levels of fear of COVID-19 had higher levels of workplace panic anxiety and workplace avoidance behavior. These findings suggest that fear of COVID-19 is an important factor that may negatively affect doctors’ mental health and consequently their work. Therefore, health administrators and authorities may have to provide sufficient resources to allay the fear of COVID-19 in addition to mentorship, an enabling working environment, and requisite mental health services.

## Data Availability

The data will be made available by the corresponding author on reasonable request.

## Abbreviations

FCV-19S: Fear of COVID-19 Scale
HCWs: Health care workers
PPE: Personal protective equipment
SOPs: Standard operating procedures

## References

[1] Jones DS (2020). History in a crisis - lessons for Covid-19. N Engl J Med.

[2] Rubin GJ, Wessely S. The psychological effects of quarantining a city. BMJ. 2020;368. 10.1136/bmj.m313. 10.1136/bmj.m313 31992552

[3] Weible CM, Nohrstedt D, Cairney P, Carter DP, Crow DA, Durnová AP, et al. COVID-19 and the policy sciences: initial reactions and perspectives. Policy Sci. 2020;53(2):225–41. 10.1007/s11077-020-09381-4. 10.1007/s11077-020-09381-4 PMC7165254 32313308

[4] Hsiang S, Allen D, Annan-Phan S, Bell K, Bolliger I, Chong T, et al. The effect of large-scale anti-contagion policies on the COVID-19 pandemic. Nature. 2020;584(7820):262–7. 10.1038/s41586-020-2404-8. 10.1038/s41586-020-2404-8 32512578

[5] Dubey S, Biswas P, Ghosh R, Chatterjee S, Dubey MJ, Chatterjee S, et al. Psychosocial impact of COVID-19. Diabetes Metab Syndr: Clin Res Rev. 2020;14(5):779–88. 10.1016/j.dsx.2020.05.035. 10.1016/j.dsx.2020.05.035 PMC7255207 32526627

[6] Ahorsu DK, Lin C-Y, Imani V, Carlbring P, Nygårdh A, Broström A, et al. Testing an app-based intervention to improve insomnia in patients with epilepsy: a randomized controlled trial. Epilepsy Behav. 2020;112:107371. 10.1016/j.yebeh.2020.107371. 10.1016/j.yebeh.2020.107371 32861897

[7] Ahorsu DK, Lin C-Y, Pakpour AH. The association between health status and insomnia, mental health, and preventive behaviors: the mediating role of fear of COVID-19. Gerontol Geriatr Med. 2020;6:2333721420966081. 10.1177/2333721420966081 PMC7594224 33195740

[8] Wang D, Hu B, Hu C, Zhu F, Liu X, Zhang J, et al. Clinical characteristics of 138 hospitalized patients with 2019 novel coronavirus-infected pneumonia in Wuhan, China. JAMA. 2020;323(11):1061–9. 10.1001/jama.2020.1585. 10.1001/jama.2020.1585 PMC7042881 32031570

[9] Baud D, Qi X, Nielsen-Saines K, Musso D, Pomar L, Favre G (2020). Real estimates of mortality following COVID-19 infection. Lancet Infect Dis.

[10] Murdoch DR, Howie SRC (2018). The global burden of lower respiratory infections: making progress, but we need to do better. Lancet Infect Dis.

[11] Spoorthy MS, Pratapa SK, Mahant S (2020). Mental health problems faced by healthcare workers due to the COVID-19 pandemic-a review. Asian J Psychiatr.

[12] Imran N, Masood HMU, Ayub M, Gondal KM. Psychological impact of COVID-19 pandemic on postgraduate trainees: A cross-sectional survey [published online ahead of print, 2020 Aug 25]. Postgrad Med J. 2020;post gradmedj-2020-138364. 10.1136/postgradmedj-2020-138364. 10.1136/postgradmedj-2020-138364 PMC7447959 32843485

[13] Atif K, Khan HU, Ullah MZ, Shah FS, Latif A. Prevalence of anxiety and depression among doctors; the unscreened and undiagnosed clientele in Lahore, Pakistan. Pak J Med Sci. 2016;32(2):294–298. doi:10.12669/pjms.322.8731 10.12669/pjms.322.8731 PMC4859009 27182226

[14] Mahase E. Coronavirus: Covid-19 has killed more people than SARS and MERS combined, despite lower case fatality rate. BMJ (Clinical research ed.), 368, m641. 10.1136/bmj.m641. 10.1136/bmj.m641 32071063

[15] Chou R, Dana T, Buckley DI, Selph S, Fu R, Totten AM (2020). Epidemiology of and risk factors for coronavirus infection in health care workers: a living rapid review. Ann Intern Med.

[16] Ali S, Noreen S, Farooq I, Bugshan A, Vohra F. Risk Assessment of healthcare workers at the frontline against COVID-19. Pak J Med Sci. 2020;36(COVID19-S4):S99-S103. doi: 10.12669/pjms.36.COVID19-S4.2790 10.12669/pjms.36.COVID19-S4.2790 PMC7306961 32582323

[17] Pappa S, Ntella V, Giannakas T, Giannakoulis VG, Papoutsi E, Katsaounou P (2020). Prevalence of depression, anxiety, and insomnia among healthcare workers during the COVID-19 pandemic: a systematic review and meta-analysis. Brain Behav Immun.

[18] Liu Q, Luo D, Haase JE, Guo Q, Wang XQ, Liu S, et al. The experiences of health-care providers during the COVID-19 crisis in China: a qualitative study. Lancet Glob Heal. 2020;8(6):e790–8. 10.1016/S2214-109X(20)30204-7. 10.1016/S2214-109X(20)30204-7 PMC7190296 32573443

[19] Kumar J, Katto MS, Siddiqui AA, Sahito B, Ahmed B, Jamil M, et al. Predictive factors associated with fear faced by healthcare workers during COVID-19 pandemic: a questionnaire-based study. Cureus. 2020;12(8):e9741. 10.7759/cureus.9741. 10.7759/cureus.9741 PMC7489766 32944456

[20] Tan Z, Khoo DWS, Zeng LA, Tien JC, Lee AKY, Ong YY, et al. Protecting health care workers in the front line: innovation in COVID-19 pandemic. J Glob Health. 2020;10(1):010357. 10.7189/jogh.10.010357. 10.7189/jogh.10.010357 PMC7242895 32509288

[21] Williamson V, Murphy D, Greenberg N (2020). COVID-19 and experiences of moral injury in front-line key workers. Occup Med (Lond).

[22] Doctors Are Struggling With Lack Of Work, Anxiety During COVID-19 Crisis, Study Finds. Pr newswire. Available at: https://www.prnewswire.com/news-releases/doctors-are-struggling-with-lack-of-work-anxiety-during-covid-19-crisis-study-finds-301057361.html. Accessed 7 Dec 2020.

[23] Muschalla B, Linden M (2014). Workplace phobia, workplace problems, and work ability among primary care patients with chronic mental disorders. J Am Board Fam Med.

[24] Urooj U, Ansari A, Siraj A, Khan S, Tariq H (2020). Expectations, fears and perceptions of doctors during Covid-19 pandemic. Pak J Med Sci.

[25] World Health Organization. COVID-19 Weekly Epidemiological Update. 2021. https://www.who.int/docs/default-source/coronaviruse/situation-reports/20210209_Weekly_Epi_Update_26.pdf. Accessed 13 Feb 2021.

[26] Atif M, Malik I. Why is Pakistan vulnerable to COVID-19 associated morbidity and mortality? A scoping review [online ahead of print]. Int J Health Plann Manage. 2020. 10.1002/hpm.3016. 10.1002/hpm.3016 PMC7404956 32700410

[27] Ahorsu DK, Lin CY, Imani V, Saffari M, Griffiths MD, Pakpour AH. The Fear of COVID-19 Scale: Development and initial validation. Int J Ment Health Addict. [online ahead of print]. 2020;1–9. 10.1007/s11469-020-00270-8. 10.1007/s11469-020-00270-8 PMC7100496 32226353

[28] Muschalla B, Linden M (2009). Workplace phobia--a first explorative study on its relation to established anxiety disorders, sick leave, and work-directed treatment. Psychol Health Med.

[29] Muschalla B, Linden M (2008). The job phobia scale. A screening instrument in medical rehabilitation. ArtzichePsychotherapie..

[30] Barua L, Zaman MS, Omi FR, Faruque M. Psychological burden of the COVID-19 pandemic and its associated factors among frontline doctors of Bangladesh: a cross-sectional study. F1000Research. 2020;9. 1304. 10.12688/f1000research.27189.3. 10.12688/f1000research.27189.1 PMC7783536 33447383

[31] Mahmood QK, Jafree SR, Qureshi WA. The psychometric validation of FCV19S in Urdu and socio-demographic association with fear in the people of the Khyber Pakhtunkhwa (KPK) province in Pakistan. Int J Ment Health Addiction. 2020. 10.1007/s11469-020-00371-4. 10.1007/s11469-020-00371-4 PMC7354742 32837443

[32] Cohen J. Statistical Power Analysis for the Behavioral Sciences. Routledge. New York; 1988.

[33] Cohen J (1992). A power primer. Psychol Bull.

[34] Korbel JO, Stegle O. Effects of the COVID-19 pandemic on life scientists. Genome Biol. 2020;21(1):113. Published 2020 May 11. 10.1186/s13059-020-02031-1. 10.1186/s13059-020-02031-1 PMC7212246 32393316

[35] Rodríguez BO, Sánchez TL. The psychosocial impact of COVID-19 on health care workers. Int Braz J Urol. 2020;46(Suppl. 1):195–200. Epub July 27, 2020. 10.1590/s1677-5538.ibju.2020.s124. 10.1590/S1677-5538.IBJU.2020.S124 PMC7719993 32618464

[36] Giorgi G, Lecca LI, Alessio F, Finstad GL, Bondanini G, Lulli LG, et al. COVID-19-related mental health effects in the workplace: a narrative review. Int J Environ Res Public Health. 2020;17(21):7857. 10.3390/ijerph17217857. 10.3390/ijerph17217857 PMC7663773 33120930

[37] Temsah MH, Al-Sohime F, Alamro N (2020). The psychological impact of COVID-19 pandemic on health care workers in a MERS-CoV endemic country. J Infect Public Health.

[38] Zhang WR, Wang K, Yin L, Zhao WF, Xue Q, Peng M, et al. Mental health and psychosocial problems of medical health workers during the COVID-19 epidemic in China. Psychother Psychosom. 2020;89(4):242–50. 10.1159/000507639. 10.1159/000507639 PMC7206349 32272480

[39] MacLeod S (2007). The challenge of providing mentorship in primary care. Postgrad Med J.

[40] Stamm M, Buddeberg-Fischer B (2011). The impact of mentoring during postgraduate training on doctors’ career success. Med Educ.

[41] Baran O, Aykac A (2020). The effect of fear of covid-19 transmission on male sexual behaviour: a cross-sectional survey study. Int J Clin Pract.

[42] IImai T, Takahashi K, Hasegawa N, Lim MK, Koh D (2005). SARS risk perceptions in healthcare workers, Japan. Emerg Infect Dis.

[43] Koh D, Lim MK, Chia SE, Ko SM, Qian F, Ng V, et al. Risk perception and impact of severe acute respiratory syndrome (SARS) on work and personal lives of healthcare workers in Singapore: what can we learn? Medical Care. 2005;(7):676–82. 10.1097/01.mlr.0000167181.36730.cc. 10.1097/01.mlr.0000167181.36730.cc 15970782

[44] Labrague LJ (2020). de los Santos, J. fear of COVID-19, psychological distress, work satisfaction and turnover intention among frontline nurses. J Nurs Manag.

